# Perceptions of Mental Disorders and Help-Seeking Behaviour for Mental Health Care Within the Maasai Community of Northern Tanzania: An Exploratory Qualitative Study

**DOI:** 10.24248/EAHRJ-D-18-00004

**Published:** 2018-11-23

**Authors:** Monica Daniel, Bernard Njau, Chauka Mtuya, Elialilia Okelo, Declare Mushi

**Affiliations:** a Department of Public Health, Kilimanjaro Christian Medical University College, Moshi, Tanzania; b Department of Psychiatry, School of Medicine, Makerere University College of Health Sciences, Kampala, Uganda

## Abstract

**Background:**

Mental disorders are rapidly becoming more prevalent worldwide and are estimated to contribute up to 15% of the global burden of disease by 2020. In Africa, the help-seeking behaviour for mental health care is complex and is hindered by misconceptions and negative attitudes towards mental disorders. This study aimed to explore perceptions of mental disorders and help-seeking behaviour for mental health care within the Maasai community in northern Tanzania.

**Methods:**

This qualitative study enrolled a purposive sample of 41 participants from a Maasai community in Arusha Region, northern Tanzania. Participants included modern health-care providers, religious leaders, traditional practitioners, local government leaders, local Maasai leaders, and workers from nongovernmental organisations dealing with mental health. Local interviewers used interview guides to conduct in-depth interviews and focus group discussions in the local language, Kiswahili. The interviews were completed between April and May 2013. We used content analysis to analyse the qualitative data.

**Results:**

Study participants attributed mental disorders to supernatural causes, such as curses, witchcraft, demons, and God's will. A few participants also mentioned biological causes and risk behaviours, including perinatal insults, head injuries, and drug abuse. Furthermore, we found that the Maasai community seeks mental health care in a sequential and simultaneous manner from 3 sectors, namely, professional health-care providers, traditional healers, and religious leaders. Traditional healers and religious leaders were preferred over professional health-care providers for the treatment of mental disorders.

**Conclusion:**

The Maasai have pluralistic help-seeking behaviour for mental health disorders. Integrating traditional healers in the modern health-care system may be beneficial to addressing mental health issues in this setting.

## INTRODUCTION

Mental disorders, defined as a combination of abnormal thoughts, emotions, behaviour, and relationships with others, contribute about 12% of the global burden of disease, and this proportion is expected to rise to 15% by 2020.^1,2^ According to the World Health Organization, more than 450 million people worldwide suffer from some form of mental disorder, most of whom live in developing countries.^2,3^ Evidence from studies conducted in different settings has shown that the contribution of mental disorders on the burden of disease differs from country to country. For example, in the United States, mental disorders are estimated to contribute 30.9% of the national disease burden, while in Finland the contribution is 32.6%.^3,4^ In Japan, mental disorders contribute about 24.6% of the burden of disease, compared to 17.6% in China and 11.6% in India.^3^ In sub-Saharan Africa, mental disorders are thought to account for a smaller proportion of the disease burden. For instance, in Namibia, mental disorders are estimated to contribute 6.9% of the burden of disease, compared to 6.6% in Togo and 5.2% in Mali.^3^ In Tanzania, mental disorders contribute about 5.3% of the disease burden.^3^ The Tanzanian Ministry of Health, Community Development, Gender, Elderly, and Children has integrated mental health in all levels of health-care service.^5–7^ Furthermore, in the Tanzanian setting, biomedical as well as traditional healers also provide primary health-care services.^8,9^ Previous studies have estimated that only 24% of people with mental disorders in Dar es Salaam attend modern health-care services compared to 48% who consult traditional healers^8^ and spiritual healers.^10^

Help-seeking behaviour refers to the sequence of problem-focused and planned remedial actions that individuals undertake to rectify perceived ill health. Help-seeking behaviour is a complex decision-making process instigated by a problem that challenges personal abilities.^11^ The process involves interpersonal interactions at the individual, family, and community levels, including biopsychosocial profiles, past experiences with health-care professionals, and the availability of alternative health-care practitioners, such as traditional or faith healers.^12,13^ Other factors that influence help-seeking behaviour include public perceptions regarding the efficiency and quality of health services and belief systems prevalent in the communities, meaning how people conceptualise the causes of health problems and how symptoms are perceived.^14–16^ The intersection between culture and help-seeking behaviour among people with mental illness is still a global concern. Sociocultural factors often influence people's decisions to delay seeking professional help or to avoid it altogether, which can, in turn, significantly compromise treatment and care.^11,16,17^

According to Kleinman's explanatory models, all cultures have systems of health beliefs to explain what causes illness, time and mode of illness onset, evolution of the illness, treatment and cure, and who should be involved in the treatment process. Specifically, the explanatory model describes a “somatic illness network”, defined as a “syndrome” of symbols and experiences, which typically “run together” for the members of a society.^14^ Mental health is a socially constructed and socially defined concept; as different societies, groups, cultures, institutions, and professionals have different ways of conceptualising its nature and causes, determining what is mentally healthy and deciding on which useful interventions, if any, are appropriate.^18,19^ It is well documented that culturally sensitive mental health-care interventions are more effective in addressing the complex spectrum of mental disorders. In fact, a goal of culturally sensitive mental health care is to specifically consider the perspectives of local cultural beliefs and practices.^20^

Explanations of mental disorders and their causes vary among individuals and communities. A study in 3 African countries – Burundi, South Sudan, and the Democratic Republic of the Congo – in 2013 showed that the perceived causes of mental disorders varied a great deal within each setting, and attributed causes varied from supernatural to psychosocial and organic. The studies showed that the perceived causes of locally defined mental disorders in these African settings were supernatural forces, natural forces, loss, and worry.^21^

A study conducted in rural Tanzania showed different patterns of explanation about mental disorders in the community.^22^ The first category was “no explanation”, whereby patients seemed to have few thoughts and explanations about why they had fallen sick and about the character of their illnesses. In the second category, the participants attributed mental disorders to spiritual causes. Finally, physical pain and reduction in daily levels of function – with physical illness presented as a central component – was the last category.

The Maasai is among the few tribes in Tanzania who prioritise culture and tradition in directing day-to-day activities and behaviours. A large proportion of the Maasai community uses traditional healers and traditional medicines to treat several illnesses. The Maasai give great respect and trust to their traditional healers, who also act as local leaders or *olaibons*.^23^ Ventevogel et al argued that life for a Maasai with a disability is particularly problematic.^21^ Throughout history, communities have reacted strongly by stigmatising, hiding, or even killing mentally and physically impaired people.^19,24^ Individuals with disabilities have faced and continue to face prejudice and discrimination in different forms – from restrictions in employment and education to sterilisation and death.^19,24^

Few studies have explored perceptions of mental disorders and help-seeking behaviours related to mental health care in Tanzania, and among the Maasai in particular. This study aimed to do just that. The findings from this study will provide insights into cultural context-specific information on perceptions of mental disorders and help-seeking behaviour for mental health care in Tanzania. The new knowledge may shed light on how the Maasai community understands and perceives mental disorders and what help-seeking behaviours they use for mental health care. The findings may assist policy makers and mental health interventionists to design culturally context-specific mental health-care interventions in the study setting.

## METHODS

### Study Area

The study was conducted in Monduli District, which is among 7 districts in Arusha Region. The district is located in the northeastern part of Tanzania. It is bordered to the north by Longido District, to the east by Arumeru District, to the south by Manyara Region, and to the west by Ngorongoro and Karatu Districts. Monduli District consists of 15 wards and 73 villages. According to the 2012 Tanzania National Census, the population of Monduli District was 158,929.^25^ The majority of people that reside in the study area are Maasai. The Maasai are a nomadic people who keep cattle, goats, and sheep. The main languages used in the district are Maasai, a local dialect, and Kiswahili, which is the national language and is spoken by most Tanzanians. Monduli District was selected because it is inhabited mainly by a Maasai community that has limited exposure to other cultures. The district has 1 public hospital, 1 health centre, and 22 dispensaries.

### Study Design

This was an exploratory community-based qualitative study conducted between April and May 2013.

### Study Population

The study population consisted of members of the Maasai community, health-care providers, religious leaders, traditional healers, local Maasai leaders, local government leaders, and nongovernmental organisations (NGOs) involved in mental health care in Monduli District.

### Sampling and Consent

The study used in-depth interviews (IDIs) and focus group discussions (FGDs) with key informants – religious leaders, traditional healers, health-care providers, local government leaders, and NGO officials – and members of the Maasai community, specifically local Maasai leaders and caretakers of patients. Purposive sampling^26,27^ was employed to recruit 21 female and 20 male participants for both IDIs and FGDs. The sampling approach was chosen to ensure the inclusion of a variety of viewpoints and diverse experiences among participants. The study participants were selected based on their involvement, knowledge, and experiences with mental disorders. We conducted an FGD for each of the following groups: caregivers, women from the general population, and men from the general population. We divided groups by gender because gender roles related to health care are very clear in Maasai culture: women are expected to take care of the sick, irrespective of the patient's gender. Additionally, we hoped the women would express themselves more freely when separated from men.^24^

All participants provided informed consent before involvement in the study. For participants who were unable to read or write, a verbal explanation was given and a thumbprint was obtained as a mark of consent. Participants were informed that participation in the study was voluntary and that they were free to withdraw at any point. Participants were assigned numbers to ensure anonymity and confidentiality; no personal identifiers were included in the collected data.

### Pretesting

The data collection tools were pretested before the study to check for clarity and internal consistency. Eight participants (4 male and 4 female) were involved in the pretest, which informed minor word use and phrasing adjustments to the final tools.

### Data Collection

Interviews with health-care providers – primarily doctors and nurses – and religious leaders were conducted in their respective offices, while the FGDs and interviews with local government leaders, local Maasai leaders, and traditional healers were all conducted in the privacy of different rooms at the village executive officer's headquarters. Interviews were conducted in Kiswahili, except for 2 IDIs that were conducted in the Maasai language for participants who did not speak fluent Kiswahili. Interviews lasted approximately 60 minutes. All interviews were recorded using a digital device, and notes were taken to capture important information. Permission to make audio recordings was obtained from study participants before commencing interviews.

### Data Management and Synthesis

Data were analysed manually using a content analysis approach. First, the verbatim transcriptions were translated into English. We then read and critically reviewed all transcripts to identify the themes, patterns, and contexts of each individual response. Using Microsoft Word, we used colour-coded highlighting and italics to identify important quotations and emerging themes from the data.

During the next stage, we organised the highlighted quotations into different categories according to the main themes that arose during the transcript analysis, while also considering the study objectives. The resulting thematic categories included inadequate knowledge, supernatural causes, biological causes, and pluralistic behaviour. We investigated and identified conceptual connections between and within categories, grouping conceptually related phenomena under the appropriate themes.^26,27^ Finally, we interpreted our findings in relation to other studies and outlined the study's implications, lessons learnt, conclusions, and recommendations. The important questions asked during the interpretation process were:

- What does the sentence or piece of data mean?
- What did the participants mean?
- What was the context of the response?
- What is the implication of the information?
- What have others written (or what is known) about it?

Representative verbatim quotations from IDIs and FGDs were selected to illustrate key findings.

### Ethical Approval

The Kilimanjaro Christian Medical University College Research and Ethics Review Committee (CRERC: No. 566) approved this study and all study activities.

## RESULTS

### Demographic Characteristics

A total of 41 participants (20 male and 21 female) were selected for the study. In total, we conducted 3 FGDs with 21 participants (7 participants per group) and 20 IDIs. About two-thirds (n=13, 65%) of the IDI participants were male, and the median age of all participants was 45 years (range, 24 to 67 years). The majority were married (n=17, 85%), had gone to school (n=14, 70%), and were Christian (n=14, 70%). The IDI participants were health-care providers (n=4), religious leaders (n=3), traditional healers (n=4), Maasai local leaders (n=4), local government leaders (n=3), and NGO officials (n=2) involved in the provision of mental health-care services in the study setting. Among the FGD participants, most were peasants (n=16, 76.2%), female (n=14, 66.7%), married (n=15, 71.4%), Christian (n=17, 80.1%), and had gone to school (n=13, 61.9%); the median age was 37 years (range, 26 to 60 years).

### Definition of Mental Disorders

The participants defined or conceptualised mental illness in different ways. Some provided spontaneous responses, while others were unable to define mental illness, although they did recall having seen someone with mental illness. Some participants equated mental illness with overt psychosis or inability to speak, while others considered mental illness as an inborn, incurable illness.

One participant said:

***I do not know what mental disorders are … But I have seen patients with mental confusion.***

(Male, Maasai leader, unknown age, IDI)

Another participant elaborated:

***Mental disorders can be associated with a person who cannot speak or is crazy.***

(Male, local government leader, 53 years old, IDI)

A female participant explained:

***Mental disorders are the diseases that if someone is born with, then it's God's work, and it cannot be treated.***

(Female, local government leader, unknown age, IDI)

### Terminology for Mental Disorders

Across interviews, participants used 4 different Maasai terms to refer to mental disorders: *ormaema* (“disabled [or abnormal] person”), *enagiormotonyi* (“disease of falling or fainting”), *ormodai* (“someone who is unable to communicate”), and *orkichaa* (“crazy person”).

### Causes of Mental Disorders

Participants mentioned several causes of mental disorders. The majority of participants ascribed mental disorders to nonmedical causes. The common nonmedical causes mentioned included supernatural powers, witchcraft, curses, and God's sickness. Other causes reported by participants included excessive alcohol use, drug abuse, and heredity. The majority of Maasai participants believed that most people with mental disorders are cursed because of their failure to observe Maasai traditions and customs, or because they had done something bad to others in the community, as one participant said:

***The cause of mental disorders in the Maasai community is believed to be witchcraft, to curses, or possession by demons.***

(Female, traditional healer, 50 years old, IDI)

Another participant stated:

***Mental disorders result from being cursed if they [patients] fail to observe traditions or elders.***

(Female, local government leader, 45 years old, IDI)

Finally, another participant emphasised the consequences of bad behaviour in the Maasai community:

***Bad behaviours, such as stealing or killing someone, can also cause mental disorders when someone is cursed.***

(Female, local government leader, unknown age, IDI)

Several participants indicated that most mental illness results from witchcraft, as a consequence of jealousy, particularly of individuals who are wealthy and successful in life.

One participant said:

***The disease [mental disorders] is caused by someone being bewitched, mostly because of the jealousy of other people.”***

(Female, local government leader, 45 years old, IDI)

Another woman said:

***The cause of the disease [mental illness] is someone being bewitched, mostly because someone has many cows, children, or wives. They give explanations like… The person that passed through the herd of cows is the one who bewitched this person.***

(Male, Maasai leader, 59 years old, IDI)

One participant described how some people believe that mental disorders can be transmitted via hostile glares. He stated:

***Being looked at by someone with a bad eye is also believed to cause mental disorders.***

(Male, 40 years old, FGD)

One participant stated that if a bird flies over a person, the shadow could cause a mental disorder:

***If someone sleeps facing upwards and a certain bird flew over that person, he or she gets enagiormotonyi.***

(Male, 32 years old, FGD).

Other causes mentioned by participants included: God's wish or punishment, stressful conditions, lack of sleep, and evil spirits. Proposed biomedical causes included incorrectly administered injections at health facilities. Interestingly, health-care providers had different perspectives on the causes of mental disorders within the Maasai community.

Participants related natural causes of illness, injuries at birth, and family history with the development of mental disorders. One participant explained:

***Mental disorders can be caused by diseases like malaria or brain injury.***

(Male, health-care provider, 45 years old, IDI)

On the other hand, a young female nurse elaborated on the complications associated with unassisted home deliveries:

***Women still give birth at home, with prolonged labour and other complications without proper care; children get brain damage and may develop mental disorders.***

(Female, health-care provider, 26 years old, IDI)

### Help-Seeking Behaviours

Substantial discussion revolved around help-seeking behaviours and beliefs related to prevention and treatment of mental disorders.

### Beliefs About Prevention

Beliefs about prevention of mental disorders revolved around traditions and customs among the study participants. Participants scarcely mentioned modern prevention strategies, as the major causes of mental disorders were thought to be related to curses, demons, and spiritual matters. The effect of the traditional mindset on beliefs about prevention of mental disorders was well illustrated by this quotation:

***Maasai believe that mental disorders cannot be prevented through modern treatment.***

(Female, NGO representative, 46 years old, IDI)

A more explicit explanation was offered by another participant:

***According to Maasai belief, in order to prevent mental disorders, people have to stop doing bad things in the community, like stealing, and should obey elders and follow traditions, and they should not engage in witchcraft.***

(Female, NGO representative, 50 years old, IDI)

### Beliefs About Treatment

Beliefs about treatment for mental disorders within the Maasai community in Monduli District were based on the same traditional mindset. Most participants believed that mental disorders could not be treated by the modern therapeutic modalities found in a hospital. Most participants explained that traditional healers, commonly known as *olaibons*, were more equipped to treat mental disorders in the Maasai community:

***People with mental disorders in our community are usually taken to olaibons, meaning traditional healers or elders in the community. This is done in order to find out what the problem is. After enquiring, traditional healers will find out if the person is cursed or bewitched, and by whom, and from there the plan to help or treat the patient is decided.***

(Female, local government leader, unknown age, IDI)

Another participant provided an alternative description of the potential healing process for a Maasai patient with mental illness:

***If the one who is believed to have cursed the patient is alive, then the elders in the community gather and take a cow and some local brew to the person who has cursed the patient to ask for forgiveness on behalf of the patient. Once the offerings are accepted, the patient will be cured. If the person refuses to forgive, it is a bad thing, then the elders either use power to make that person forgive, or they also curse that person.***

(Male, Maasai leader, 63 years old, IDI)

Use of different paraphernalia was also mentioned as part of the alternative healing practices for mentally ill patients in the Maasai community:

***Also, to cleanse the curse, the elders spit on a rope called ngopito and tie the rope on the patient and do special prayers to break the curse. If the person who triggered the curse has died, elders visit the grave and give offerings to ask for forgiveness and recite a special prayer.***

(Male, traditional healer, 55 years old, IDI)

Across interviews, participants emphasised the difficulty involved in curing mental disorders caused by supernatural forces. One participant said:

***For those bewitched, it is hard to treat in the community; it takes time to be healed.***

(Male, religious leader, 55 years old, IDI)

Spiritual prayers were another reported treatment option for mental disorders. A male religious priest said:

***When those kinds of people – with mental disorders – are brought here, first I baptise them, followed by prayers, and the patient is told to affirm that they are cured and turn their beliefs and lives to God. We apply water, olive oil, and a type of juice called fruto [a blackcurrant juice], which we pray upon to turn it [the juice] into the blood of Jesus for the patient to drink. Thereafter, the patient is cured, or if not, the Holy Spirit will show me what else to do. Normally, we tell them never to sin again and to fear God.***

(Male, religious leader, 24 years old, IDI)

Spiritual leaders reported conflicting beliefs about what should be done after prayers, once the patient is declared healed. The main point of contention was whether the patient should seek help at a hospital after the prayers or not.

Another treatment option believed to cure mental disorders among the Maasai in this community was the use of herbs. Ambiguity was centred on whether herbs alone could treat mental disorders, or if they should be used in conjunction with other treatment options. The uncertainty was well elaborated by a female caregiver:

***Treating patients with mental disorders is not easy by using herbs, but we try; and when they fail, we refer patients to either traditional healers, spiritual healers for prayers, or the hospital.***

(Female, caregiver, unknown age, FGD)

An older female herbalist reported:

***When a patient of that type [with a mental disorder] is brought to me, I make small cuts [scarifications] on the body and apply herbs on the wounds. I examine the patient and provide first aid. I massage the whole body, especially the stomach and hands. When massaging the body, I realise where the problem is and if it can be cured or not. After examining the patient and after first aid, if I discover that I cannot treat the disease, I refer the patient to wherever they can be treated, either by olaibons, or the hospital, or prayers.***

(Female, traditional healer, unknown age, IDI)

### Preferred Treatment for Mental Disorders

Participants generally preferred traditional treatment methods. A female herbalist said:

***Most patients go to traditional healers because they believe that they are bewitched… and get cured.***

(Female, traditional healer, 50 years old, IDI)

Some participants indicated a strong aversion to modern therapies for mental disorders:

***People prefer traditional healers because they believe them to be more effective than modern medicine; however, after other treatment options fail, then the patient is taken to the hospital as the last option.***

(Male, 55 years old, FGD)

During IDIs, most participants mentioned that modern therapies are regarded as the last option, once the traditional treatment options fail.

***They believe that traditional medicine can cure mental disorders; hospital treatment is the last option.***

(Male, health-care provider, 55 years old, IDI)

Another perspective took educational status into account:

***Educated relatives are more likely to seek treatment from the hospital, while the uneducated will start with herbs or traditional healers.***

(Female, caregiver, 59 years old, FGD)

## DISCUSSION

This study aimed to explore perceptions of mental disorders and help-seeking behaviour related to mental health among the Maasai people in Arusha Region. The study findings demonstrated a range of misconceptions about mental health within this community. The causes of mental disorders were attributed to either sociocultural and spiritual (eg, curses, bewitching, or hostile glaring) or biological (eg, disease or heredity) factors. This Maasai community perceived a pluralistic framework for help-seeking behaviour and preferred traditional health care over modern health-care services.

Having correct knowledge of the causes of and potential treatments for mental illness is important because this can influence positive help-seeking behaviour and appropriate care. In this study, participants explained mental disorders using both social and biomedical models.^14^ From the social-model perspective, participants had a number of misconceptions about the causes of mental disorders. They attributed mental disorders to supernatural experiences, such as God's will, curses, witchcraft, and antagonistic stares. These results support previous findings from studies conducted in Tanzania in Kinondoni District in Dar es Salaam^28^ and Mbulu District in Manyara Region,^22^ as well as studies conducted in Vietnam^21^ and Nigeria.^29^

In Maasai society, curses or acts of God are attributed to shameful or harmful behaviour on the part of figures of authority or respect, such as parents or grandparents.^24^ In this study, Maasai participants also believed that mental illness can result from bad deeds or crimes leading to the cursing or bewitching of the perpetrator. These beliefs about the causes of mental disorders can lead to poor disease management, leaving many patients untreated and increasing the burden of mental illness in the community.

In this study, some participants attributed mental disorders to biomedical causes, including genetics and peri-natal injury. Concerning hereditary factors, some participants rightly stated that family history can predispose individuals to mental illness; however, despite some understanding of the biomedical contribution to mental disease causes, participants often ascribed the hereditary component of mental illness to a “family curse”. Studies in Uganda and Sri Lanka have shown that families with a history of mental illness are often stigmatised and discriminated against.^30,31^

Other illnesses, malaria in particular, were reported by participants to be associated with mental disorders, although they could not give a plausible explanation on how malaria can cause mental illness. According to Idro et al, cerebral malaria may predispose children to long-term mental disorders, possibly due to convulsions, impaired consciousness, and coma, leading to brain damage.^32^ Furthermore, injuries to neonates during unassisted childbirth were perceived by participants to cause mental illness. Evidence suggests that children with birth injuries often develop mental illness in adulthood.^33^

Generally, the Maasai participants in our study believed that people cannot acquire mental disorders without an identifiable cause and that most cases are due to supernatural causes, often facilitated by human actions or behaviours. These findings are in line with other studies.^22,24,28,29,34^ However, key informants were of the opinion that these kinds of beliefs were slowly changing over time in the Maasai community.

According to Jain et al,^35^ help-seeking behaviour, particularly in rural communities, is a complex outcome of many factors operating at the individual, family, and community levels, including biosocial factors, an individual's past experiences with health services, and the availability of alternative health care.

In describing help-seeking behaviour, Kleinman's explanatory models help describe what influences patients to navigate through different therapeutic options. The explanatory models in this context focus on the treatment options that are employed by all those engaged in the clinical process. Kleinman further argues that explanatory models are held by both patients and practitioners, that these models offer explanations of sickness and treatment to guide choices among available therapies and therapists and cast personal and social meanings on the experience of sickness.^14^

Based on this framework, in this study, we found that the Maasai have 3 major treatment options for mental health concerns, namely, modern health-care services, traditional care, and spiritual care. In most instances, patients and relatives will use 1 or more treatment options at a time, depending on the perceived causes of illness and the cost and effectiveness of care. The 3 pathways describe the conceptualisation of help-seeking behaviour for mental disorders within the Maasai community in sequential patterns that are influenced by factors, such as social norms (eg, beliefs about causes of disease) and literacy levels. The first pattern was to first seek assistance from traditional healers, then spiritual healers, and, finally, modern health-care providers. The second pattern was to seek assistance from herbalists, and then, in order, traditional healers, spiritual healers, and modern health-care providers. The third pattern was to first seek help from modern health-care providers, then traditional healers, and, finally, spiritual healers.

Most participants in this study believed that mental disorders can be prevented by observing some cultural traditions and practices, stopping bad behaviours, and preventing or refraining from witchcraft. Several studies on mental disorders and other chronic diseases have reported similar perceptions.^29,36^

Most participants believed that traditional healers should be the main treatment providers for mental disorders and that biomedical personnel either should not be consulted or should be considered as the last option.^8,30,36^ This study also revealed that traditional healers perform physical examinations and some forms of massage as part of their mental health assessment and therapeutic procedures. Traditional healers were reported to look beyond physical treatment of illness by trying to deal with perceived causes, such as curses and witchcraft.^37^

Although the majority of participants believed that mental disorders could be treated using alternative medicine, only a few participants had confidence in modern health-care services. Participants who believed in modern health-care services were often those with some formal education or were health-care providers themselves. The study also found that some Maasai community members depend on spiritual prayers. Participants reported that during prayers, holy water, blessed fruit juice, Bible quotations, or self-confessions were used to break curses and to cast out demons. This is in line with a study reported by Atindanbila and Thompson,^37^ which demonstrated that spiritual treatment for people with mental disorders is common and that spiritual healers use confession, biblical or Koranic quotations, laying of hands, holy water, and salt when dealing with mental disorders.

An interesting finding was that some religious leaders who held dual beliefs were reported to have advised patients to attend modern health-care services for further treatment, while others reportedly believed that prayers were enough. Furthermore, some religious leaders, mainly Christians, did not allow their followers to go to traditional healers, including the Maasai elders, or *olaibons*.

### Limitations

This study had some important limitations. The first limitation was language. Some participants were not conversant with Kiswahili, the language widely used in Tanzania. To rectify the language limitation, a research assistant fluent in the Maasai language translated the interviews and responses from Maasai to Kiswahili. The second limitation was related to the sensitive subject matter. Mental disorders are widely associated with stigma, and this might have prevented participants from freely discussing matters pertaining to mental disorders, particularly during the FGDs. Efforts to overcome this limitation included spending considerable time with participants to build rapport before conducting the interviews. The aim was to make participants feel comfortable and relaxed. In addition, the interviewers used probing and prompting questions or statements during the IDIs and FGDs to reduce any stigma or discomfort associated with discussing mental disorders. The final limitation is an inherent weakness of qualitative study designs, whereby the findings may not be representative of the population in the study setting much less other settings. Because the study participants were recruited from a rural setting, with the majority of participants being from the Maasai community, the study findings may not be relevant to urban settings or non-Maasai communities. Despite these limitations, the study findings do contribute knowledge about perceptions of mental disorders and help-seeking behaviour for mental health among the Maasai in this region. The study findings could form a basis for further mental health studies among Maasai and other pastoral communities.

## CONCLUSION

This study explored the perceptions of mental disorders and help-seeking behaviour for mental health care among Maasai in Monduli District, Arusha Region. In several ways, the current study corroborated what has previously been reported and added new knowledge, particularly on how mental illness is conceptualised and the culture-specific and cognitive factors influencing help-seeking behaviour in a Maasai community.

Knowledge about the causes and treatment of mental disorders within this Maasai community were mixed. The Maasai have both naturalistic and personalist views of mental health, as they associate mental illness with both biology and witchcraft, curses, and punishment from God. Secondly, patients and family members have multiple options for seeking care, with the majority of people using traditional healers and spiritual healers more than modern health-care providers.

These findings have 2 key primary health-care implications on mental health in this community. Firstly, they underline the urgency for advocacy initiatives that may increase mental health literacy and awareness at the individual, family, and community levels. The advocacy initiatives should emphasise the importance of the social cognitive factors reported in this study that influence help-seeking behaviour. Secondly, the findings call for interventions that will empower traditional healers to identify people with mental disorders and participate in the design of mental health prevention programmes in this study setting.
